# Case Commentary: Intraventricular polymyxin B—small steps, big questions

**DOI:** 10.1128/aac.01527-25

**Published:** 2026-01-27

**Authors:** Nitin Das Kunnathu Puthanveedu, Adarsh Bhimraj

**Affiliations:** 1 Section of Infectious Diseases, Department of Internal Medicine, University of Illinois College of Medicine 221336, Peoria, Illinois, USA; 2 Division of Infectious Diseases, Department of Internal Medicine, Houston Methodist Hospital 828032, Houston, Texas, USA; Houston Methodist Hospital, Weill Cornell Medical College, Houston, Texas, USA

**Keywords:** ventriculitis, polymyxins, intraventricular therapy, healthcare-associated ventriculitis and meningitis, HCAVM, pharmacokinetics

## Abstract

Carbapenem-resistant gram-negative ventriculitis is a life-threatening infection with limited treatment options. H. Jiang, Y. Hu, J. Cai, J. Zhang, et al. (Antimicrob Agents Chemother 70:e00943-25, 2026, https://doi.org/10.1128/aac.00943-25) describe two patients with this condition who were successfully treated with intraventricular polymyxin B. Their report highlights uneven antibiotic distribution within the ventricles, the influence of cerebrospinal fluid drainage on intraventricular drug concentrations, and cure with intraventricular polymyxin B alone. These findings raise important questions about optimal intraventricular antimicrobial dosing and whether concurrent intravenous therapy is needed in healthcare-associated ventriculitis.

## COMMENTARY

In this issue, Jiang and colleagues (1) describe two patients with cerebral ventriculitis due to carbapenem-resistant gram-negative bacilli (CR-GNB) who were successfully treated with intraventricular polymyxin B. Clinical and microbiologic cure was achieved, and serial cerebrospinal fluid (CSF) parameters and drug concentrations were characterized.

Increasing antimicrobial resistance in gram-negative pathogens, particularly to carbapenems, poses a major clinical challenge in modern practice. One retrospective study demonstrated that the in-hospital mortality rate of healthcare-associated gram-negative meningitis in the carbapenem-non-susceptible group was more than double the rate in the carbapenem-susceptible group (18.8% vs 7.4%, *P* = 0.001) (2).

A key challenge in these patients is that intravenous antimicrobials may not achieve optimal CSF pharmacokinetic/pharmacodynamic (PK/PD) endpoints due to poor penetration across the blood-CSF barrier and high minimum inhibitory concentration values. This is particularly problematic for CR-GNB, for which the beta-lactams routinely used to treat cerebral ventriculitis, such as meropenem or cefepime, are ineffective. Alternatives such as intravenous polymyxins and aminoglycosides achieve limited CSF drug exposures and are limited by a narrow therapeutic window. Use of newer agents, such as ceftazidime-avibactam and cefiderocol, is still nascent, with very limited clinical data in central nervous system infections (3). Intraventricular antimicrobial therapy (IVT) is considered in these scenarios where infection is difficult to eradicate with intravenous antimicrobial therapy alone (4). Intraventricular antimicrobial administration achieves high CSF drug exposures without excessive systemic toxicity (5, 6). A 2018 meta-analysis found that the benefit of combined IVT and IV therapy, compared with IV therapy alone for gram-negative healthcare-associated ventriculitis and meningitis (HCAVM), was essentially confined to the CR-GNB subgroup (overall mortality OR, 0.22; 95% CI, 0.08–0.60). The pathogen eradication rate and infection-associated mortality were also better in this subgroup (7).

Polymyxins still have a role in the treatment of MDR gram-negative ventriculitis, as susceptibility to this class is relatively preserved in these pathogens (8–10). Intravenous polymyxins can cause nephrotoxicity, neurotoxicity, and have poor CSF penetration due to high polarity. Intraventricular polymyxin B achieves higher CSF concentrations and has been associated with higher microbiological cure rates and reduced 30-day mortality in patients with HCAVM due to CR-GNB (8). Yet several questions remain unanswered: What is the optimal dose and dosing interval? Can imaging-based estimates of ventricular volume be reliably used to estimate dosing? To what extent does external ventricular drain (EVD) output affect drug clearance from the CSF? Do drug concentrations equilibrate reliably between the cerebral ventricles?

In this context, the report by Jiang and colleagues offers several key insights. Prior PK studies of IVT and intrathecal therapy have noted substantial differences in drug concentrations between lumbar subarachnoid CSF and ventricular CSF (11). This two-case series demonstrates that intraventricular drug administration may not lead to homogenous drug distribution even within the ventricles. In a prior case report, an adult treated with intraventricular daptomycin for EVD-associated ventriculitis also had higher CSF drug concentrations in the ventricle on the dosing side than in the contralateral ventricle (12). This may reflect loculated CSF compartments or poor communication between the ventricles due to inflammation.

Jiang and colleagues explore how the rate of CSF drainage affects intraventricular drug clearance and suggest that reduced CSF drainage increases intraventricular drug retention. This makes intuitive sense, and the 2017 Infectious Diseases Society of America HCAVM guidelines cite a previously proposed dosing schedule for intraventricular antimicrobials based on CSF drainage volume, but it still requires validation in clinical studies (4).

It is also noteworthy that both patients received only intraventricular polymyxin B, without systemic antimicrobials, and still achieved microbiologic and clinical cure. Similarly, one small trial randomized patients with EVD-associated ventriculitis to receive vancomycin either by the intravenous route or by the intraventricular route. Clinical and microbiological cure was achieved within similar time periods in both groups (6). These reports suggest that there may be cases of HCAVM where intraventricular antimicrobials alone may be sufficient to treat the infection, but the data are still very limited.

Overall, the findings by Jiang and colleagues may be viewed as hypothesis-generating rather than practice-changing, raising questions such as:

- Is intraventricular therapy alone sufficient in selected cases of ventriculitis?
- Should the intraventricular antimicrobial dosing interval be based on EVD CSF output?
- In patients with bilateral EVDs, should the drug be given through both EVDs?

Prospective PK/PD studies that relate key drug exposure indices to microbiological and clinical outcomes are needed to answer these questions.
